# A Novel Mouse Model of Advanced Diabetic Kidney Disease

**DOI:** 10.1371/journal.pone.0113459

**Published:** 2014-12-16

**Authors:** Jean-Francois Thibodeau, Chet E. Holterman, Dylan Burger, Naomi C. Read, Timothy L. Reudelhuber, Christopher R. J. Kennedy

**Affiliations:** 1 Kidney Research Centre, Division of Nephrology, Ottawa Hospital Research Institute, Ottawa, Ontario, Canada; 2 Department of Cellular and Molecular Medicine, Faculty of Medicine, University of Ottawa, Ottawa, Ontario, Canada; 3 Clinical Research Institute of Montreal, University of Montreal, Montreal, Quebec, Canada; University of Tokushima, Japan

## Abstract

Currently available rodent models exhibit characteristics of early diabetic nephropathy (DN) such as hyperfiltration, mesangial expansion, and albuminuria yet features of late DN (hypertension, GFR decline, tubulointerstitial fibrosis) are absent or require a significant time investment for full phenotype development. Accordingly, the aim of the present study was to develop a mouse model of advanced DN with hypertension superimposed (HD mice). Mice transgenic for human renin cDNA under the control of the transthyretin promoter (TTRhRen) were employed as a model of angiotensin-dependent hypertension. Diabetes was induced in TTRhRen mice through low dose streptozotocin (HD-STZ mice) or by intercrossing with OVE26 diabetic mice (HD-OVE mice). Both HD-STZ and HD-OVE mice displayed more pronounced increases in urinary albumin levels as compared with their diabetic littermates. Additionally, HD mice displayed renal hypertrophy, advanced glomerular scarring and evidence of tubulointerstitial fibrosis. Both HD-OVE and HD-STZ mice showed evidence of GFR decline as FITC-inulin clearance was decreased compared to hyperfiltering STZ and OVE mice. Taken together our results suggest that HD mice represent a robust model of type I DN that recapitulates key features of human disease which may be significant in studying the pathogenesis of DN and in the assessment of putative therapeutics.

## Introduction

Diabetic nephropathy (DN) is a serious microvascular complication that affects a significant proportion of patients suffering from both type 1 and type 2 diabetes, accounting for over 40% of end-stage renal disease (ESRD) cases in North America [1]. Current interventions that target the renin-angiotensin aldosterone system (RAAS) along with strict glycemic control are associated with a slower deterioration of renal function and delayed ESRD onset in patients with diabetes. However, these therapies only slow progression and do not cure the disease [2]. Thus a pressing issue remains the development of new treatment strategies.

Research focused on novel therapeutic interventions for the treatment of DN has been significantly hindered by the fact that animal models fail to reliably recapitulate the full spectrum of human disease. In 2005 the National Institute of Diabetes and Digestive and Kidney Diseases (NIDDK) established the Animal Models of Diabetic Complications Consortium (AMDCC) with the objective of developing a list of criteria for validating progressive DN in mouse models [3]. These criteria were further updated in 2009 and provide a benchmark against which current DN models are measured [4]. As reviewed elsewhere, the majority of mouse models currently available develop pathologies reminiscent of early DN provided they are bred onto susceptible backgrounds [5], [6], [7], [8], [9]. However changes associated with advanced DN such as tubulointerstitial fibrosis, arteriolar hyalinosis, and >50% decline in GFR over the lifetime of the animal are often absent. A limited number of mouse models do meet the majority of AMDCC criteria, such as the *eNOS* ^−/−^ *db/db* and BTBR *ob/ob* models, however the complex breeding strategies and significant time investment required for the pathological changes to develop are restrictive. Therefore we sought to develop a new mouse model that would rapidly develop pathological changes associated with advanced DN while being tractable to genetic manipulation.

In this study we have employed transgenic mice with the human renin cDNA under the control of the transthyretin promoter (TTRhRen) and induced diabetes either through streptozotocin (STZ)-injections or by crossing with the OVE26 transgenic type 1 diabetes mouse on the susceptible FVB/n background. These mice consistently display features of advanced DN outlined by the Diabetes Complications Consortium including >10-fold increase in albuminuria, mesangial matrix expansion, tubulointerstitial fibrosis, and signs of GFR decline [4]. These animals are amenable to the current array of genetic strategies (i.e., gene-targeting/transgenics) that are used widely to explore the role of any number of putative players in the progression of DN.

## Results

### Systolic BP is progressively increased in HD mice

Two models of HD mice were studied. In the first model, 8–12 week-old male WT and TTRhRen mice were subjected to a low-dose STZ diabetes regimen (HD-STZ) and followed for 18 weeks. For the second model, OVE26 and TTRhRen mice were intercrossed to obtain HD-OVE mice, the males of which were followed for up to 20 weeks of age. Cardiac and renal hypertrophy were analyzed by normalizing kidney and heart weights to tibia length. (Tables 1 and 2). Similar plasma glucose levels were measured for both HD-STZ and HD-OVE26 models (STZ study: WT, 10.5±1; H, 12.1±1; STZ, 30.7±2; HD-STZ, 31.2±2 and OVE26 study: WT, 11.3±1; H, 12.4±1; OVE, 32.8±2; HD-OVE, 35±0 mM). In addition, decreased bodyweight was noted in OVE26 mice. Characteristic renal hypertrophy accompanied the hyperglycemia in both STZ and OVE cohorts, while HD-OVE blood glucose values were slightly albeit significantly higher than OVE mice. Non-diabetic hypertensive mice did not develop renal hypertrophy, but showed a non-significant trend towards increased heart-to-tibia ratios.

**Table 1:** OVE26 study physiological parameters and organ hypertrophy.

	WT	H	OVE	HD-OVE
Plasma glucose (mg/dL)	11.3±0.7	12.3±1	29.9±0.8*	35± N/A**^†‡^**
Bodyweight (g)	32.4±1.2	32.3±1.1	27.3±0.9*	26.4±1.1**^†^**
Right kidney/tibia (mg/mm)	12.3±1.3	10.8±0.7	17.1±1.4*	25.6±5.6**^†‡^**
Heart/tibia (mg/mm)	8.4±0.1	9.3±0.6	7.3±0.5	8.6±0.7

* = P≤0.01 vs. WT; **^†^** = P≤0.01 vs. H; **^‡^** = P≤0.05 vs. OVE.

**Table 2:** STZ study physiological parameters and organ hypertrophy.

	WT	H	STZ	HD-STZ
Plasma glucose (mg/dL)	10.5±0.8	12.1±0.5	30.8±1.7	31.2±1.9
Bodyweight (g)	28.8±1.7	32.8±1.5	30.7±1.1	33.5±1.1
Right kidney/tibia (mg/mm)	10.3±0.2	13.1±0.6**^†^**	17.4±1.1*	14.5±1.3**^‡^**
Heart/tibia (mg/mm)	7.1±0.3	9.7±0.1	8.7±0.5	9.7±0.6

* = P≤0.01 vs. WT; **^†^** = P≤0.05 vs. WT; **^‡^** = P≤0.05 vs. STZ.

**Figure 1:**
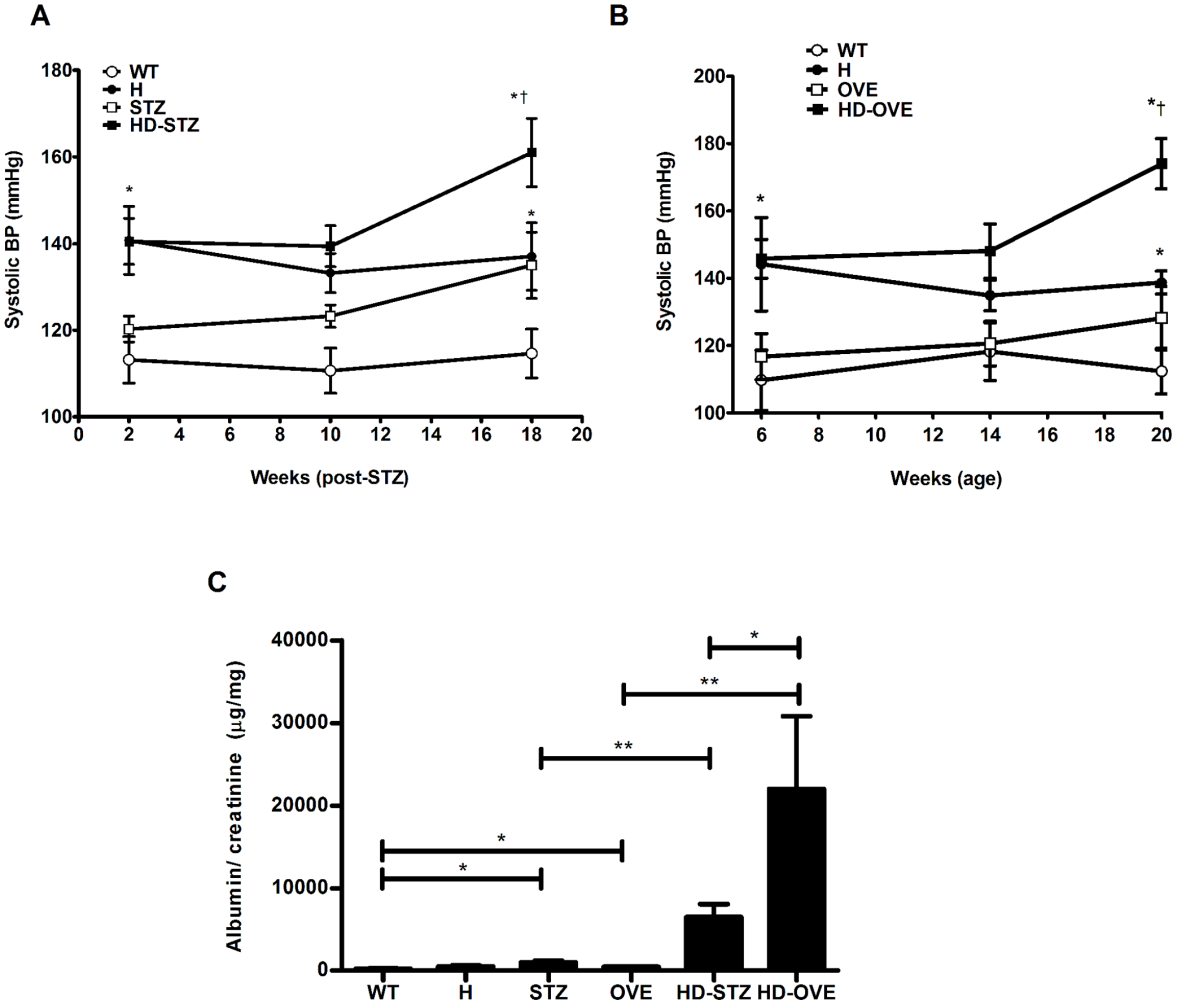
Systolic BP and albuminuria. Longitudinal BP measurements were obtained by tail-cuff plethysmography (A,B) while urinary ACR levels were measured in urine samples at endpoint (C) using an ELISA-based method (Bethyl) in both the STZ (4–6 mice per group) and OVE26 studies (n = 4–7 mice per group). Data represented as mean with standard error. (* = P≤0.05; ^†^ = P≤0.05 vs. WT control).

Longitudinal systolic BP was assessed throughout the study in both models (Fig. 1a–b). We observed equivalent BP elevations for H and HD-STZ groups 2 weeks post-STZ, (WT, 113±5; H, 140±8; STZ, 120±3; HD-STZ, 140±5 mmHg). These values increased progressively and significantly in the HD-STZ group, and to a lesser degree in the STZ mice, while H mice showed a slight reduction at 18 weeks post-injection (WT, 114±6; H, 137±8; STZ, 135±7; HD-STZ, 161±7 mmHg). In the HD-OVE study (Fig. 1b), baseline (6 weeks of age) BP was elevated in H and HD-OVE mice versus WT and OVE mice (WT, 109±9; H, 144±13; OVE, 116±7; HD-OVE, 145±5 mmHg). The combination of both hypertension and diabetes led to a persistent and significant rise in BP that significantly exceeded that of H mice by 20 weeks of age (WT, 112±7; H, 138±3; OVE, 128±9; HD-OVE, 174±7 mmHg).

### Exacerbated albuminuria in HD mice

In order to examine the effects of hypertension superimposed upon diabetes on filtration barrier integrity, urine albumin-to-creatinine ratios (ACR; *µg/mg*) were determined (Fig. 1c). Increased ACR levels were observed in STZ-treated mice, while the HD-STZ phenotype exacerbated this parameter. In the HD-OVE model, hypertension alone did not lead to albuminuria, while diabetes led to a significant 3-fold increase in ACR versus WT. Remarkably, at 20 weeks of age HD-OVE mice exhibited a 40-fold increase in ACR versus OVE mice, suggesting significant glomerular filtration barrier dysfunction (WT, 245±69; H, 504±166; STZ, 1026±204; OVE, 483±81; HD-STZ, 6504±1584; HD-OVE, 22023±4802 µg/mg, ACR µg/mg).

### Glomerular hypertrophy and mesangial matrix expansion is exacerbated in HD mice

**Figure 2:**
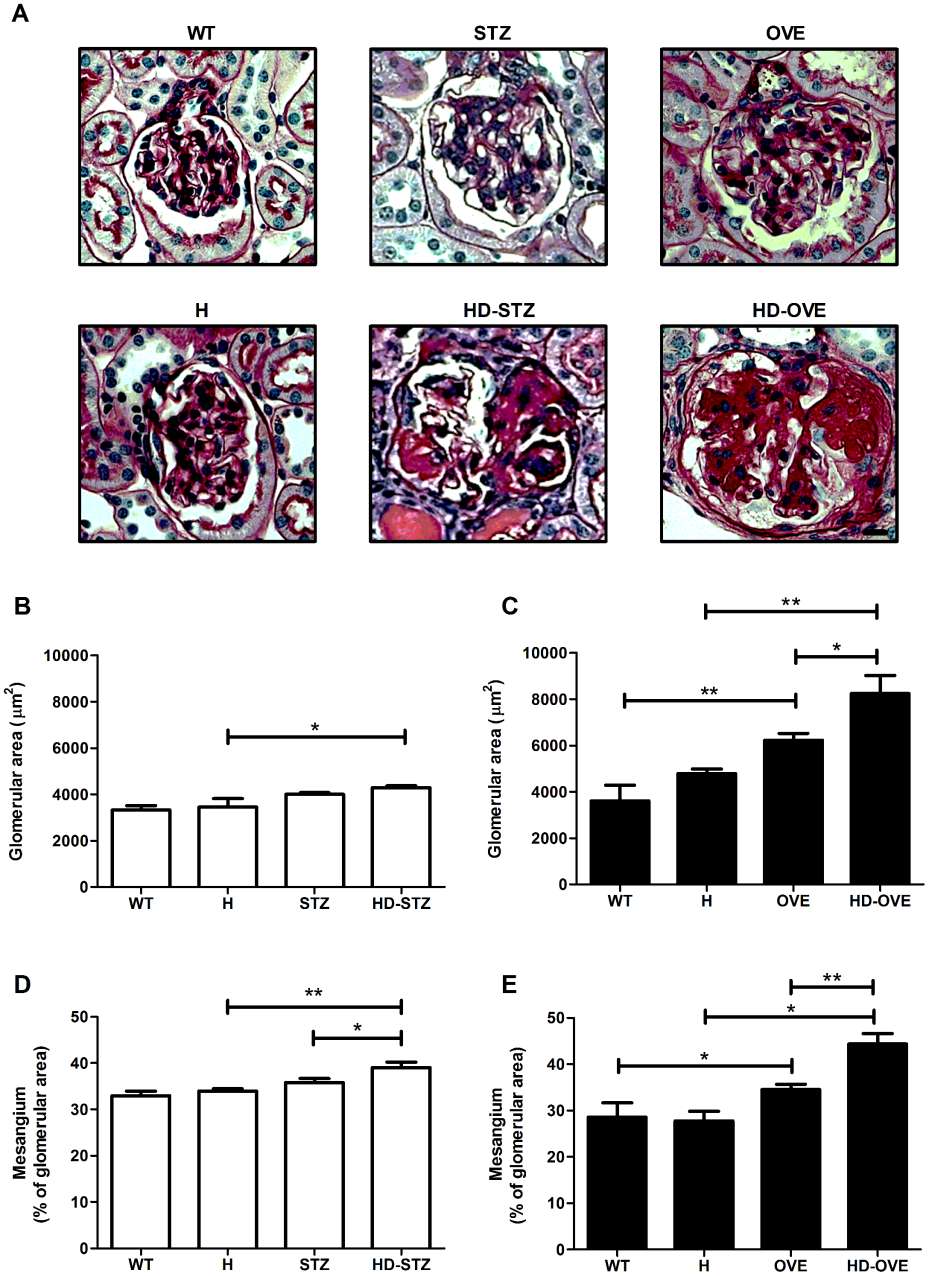
Glomerular pathology. Paraffin-embedded PFA fixed-kidney sections were stained with periodic-acid Schiff. (A) Representative images of glomerular profiles for each group. Glomerular surface area (B, C) and mesangial area (D, E) analysis was performed on 15–25 glomeruli per mouse, 3–5 mice per group. Data represented as means with standard error. * = P≤0.05; ** = P≤0.01. (Scale bar = 5 um, 40X Mag.).

Persistent hyperglycemia leads to glomerular hypertrophy and induces mesangial matrix overproduction. We analyzed glomerular profiles from both HD-STZ and HD-OVE cohorts (Fig. 2). While the onset of hypertension yielded observable increases in glomerular surface area, these levels were significantly surpassed in the HD-STZ mice and greatly exceeded that of STZ mice (WT, 3321±191; H, 3442±370; STZ, 3996±78; HD-STZ, 4281±87 µm^2^). Similar findings were obtained for the HD-OVE (WT, 3601±638; H, 4778±201; OVE, 6223±300; HD-OVE, 8235±785 µm^2^). Accordingly, mesangial area as a percentage of total glomerular surface area was also increased in diabetic mice from both studies, which was worsened when hypertension was present (STZ study: WT, 32.9±1; H, 33.8±1; STZ, 35.7±1; HD-STZ, 39±1 and OVE26 study: WT, 28.6±3; H, 27.7±2; OVE, 34.5±1; HD-OVE, 44.4±2, % of glomerular area). Furthermore, the presence of proteinaceous material in the tubules of HD-OVE mice is consistent with compromised glomerular structural integrity in this group.

### Renal tubulointerstitial fibrosis and elevated α-SMA in HD-OVE mice

**Figure 3:**
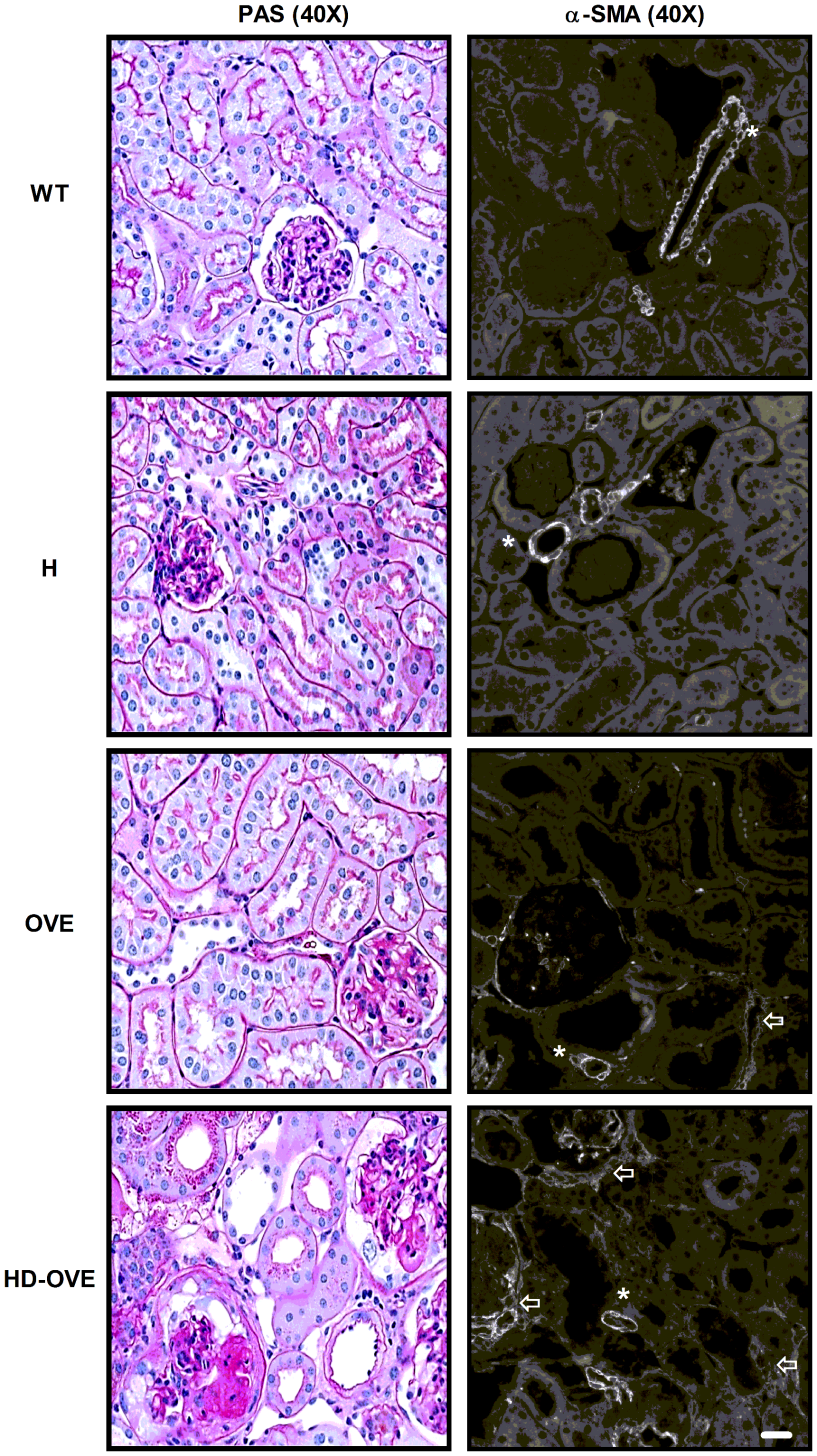
OVE26 study - PAS and α-SMA staining. Paraffin-embedded PFA fixed-kidney sections were stained with periodic-acid Schiff (left) or α-SMA (right) and visualized by either light or fluorescence microscopy at 40X. Representative images. (Scale bar = 10 um).

The impact of the HD phenotype on fibrosis of the kidney’s tubulointerstitium was examined in a qualitative manner. Using microscopic examination, increased PAS-positive material was observed in most HD-OVE mice compared to uniquely diabetic counterparts. In contrast to the OVE26 study, while in agreement with the STZ model’s characteristic milder phenotype, a portion of HD-STZ mice showed some signs of interstitial damage yet to a lesser extent than the HD-OVE cohort (data not shown). Under immunofluorescence microscopy, enhanced immunodetectable α-SMA was evident in both the interstitium and in peri-glomerular areas (crescentic glomerulosclerosis) for the HD-OVE cohort (*arrows*, Fig. 3), while similar baseline vascular α-SMA staining was observed in all mice (*asterisks*, Fig. 3).

### Increased collagen and fibronectin production in HD-OVE mice

**Figure 4:**
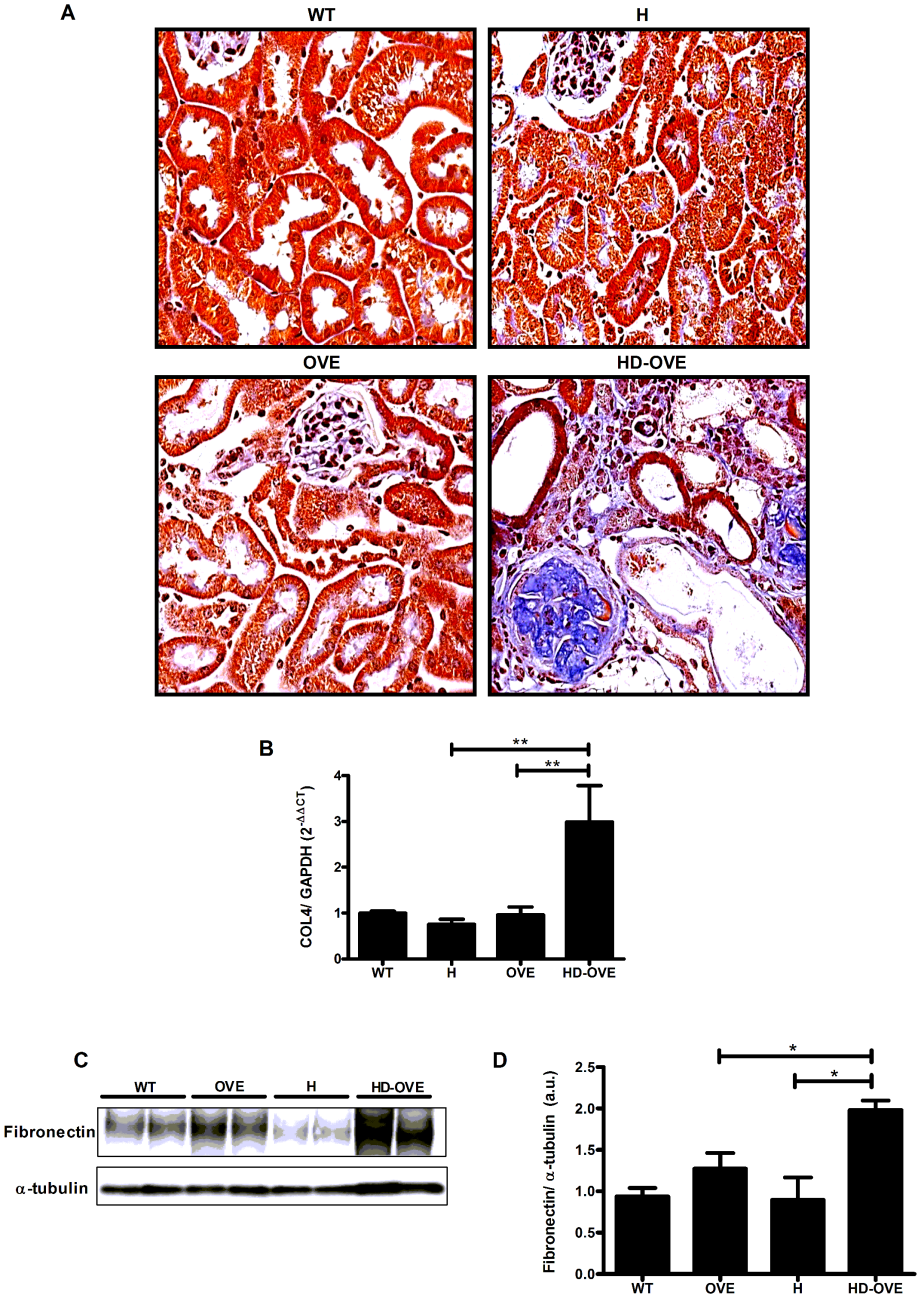
OVE26 study - collagen and fibronectin expression. A) Representative images of paraffin-embedded PFA fixed-kidney sections stained with Masson’s trichrome (40X mag.) B) qPCR determination of collagen-4 mRNA expression in kidney cortex normalized to GAPDH (n = 3–5 mice/group). C) Representative fibronectin and α-tubulin protein immunoblotting in kidney cortex samples. D) Quantification of fibronectin expression in OVE26 study kidney cortex. (n = 4–6 mice/group; * = P≤0.05; ** = P≤0.01).

Further understanding of the HD-OVE cohort’s propensity for developing advanced glomerular and tubulointerstitial lesions earlier than their OVE littermates was confirmed using Masson’s trichrome staining on kidney sections (Fig. 4a). Positive staining for collagen (in blue) was readily observed in the glomerular tuft and in the tubulointerstitial regions of HD-OVE kidneys, while being minimally increased in OVE mice and absent from H and WT groups. To confirm increased collagen expression, we measured collagen-4 mRNA levels by qPCR of kidney cortex RNA isolates. Accordingly (Fig. 4b), HD-OVE mice harbored a three-fold increase in collagen-4 mRNA levels versus WT, H or OVE alone (WT, 0.99±0.04; H, 0.75±0.11; OVE, 0.96±0.17; HD-OVE, 2.99±0.8, a.u.). Immunoblotting for fibronectin was also performed in cortical lysates from the OVE study (Fig. 4c). H and OVE mice exhibited similar fibronectin protein levels as WT controls. However HD-OVE mice showed greater increases fibronectin production (Fig. 4d.) (WT, 0.93±0.1; OVE, 1.3±0.2; H, 0.90±0.2; HD-OVE, 1.9±0.1, a.u.), corroborating the indications of tubulointerstitial fibrosis and the increases in α-SMA protein observed by immunofluorescence.

### Decreased GFR in HD mice

**Figure 5:**
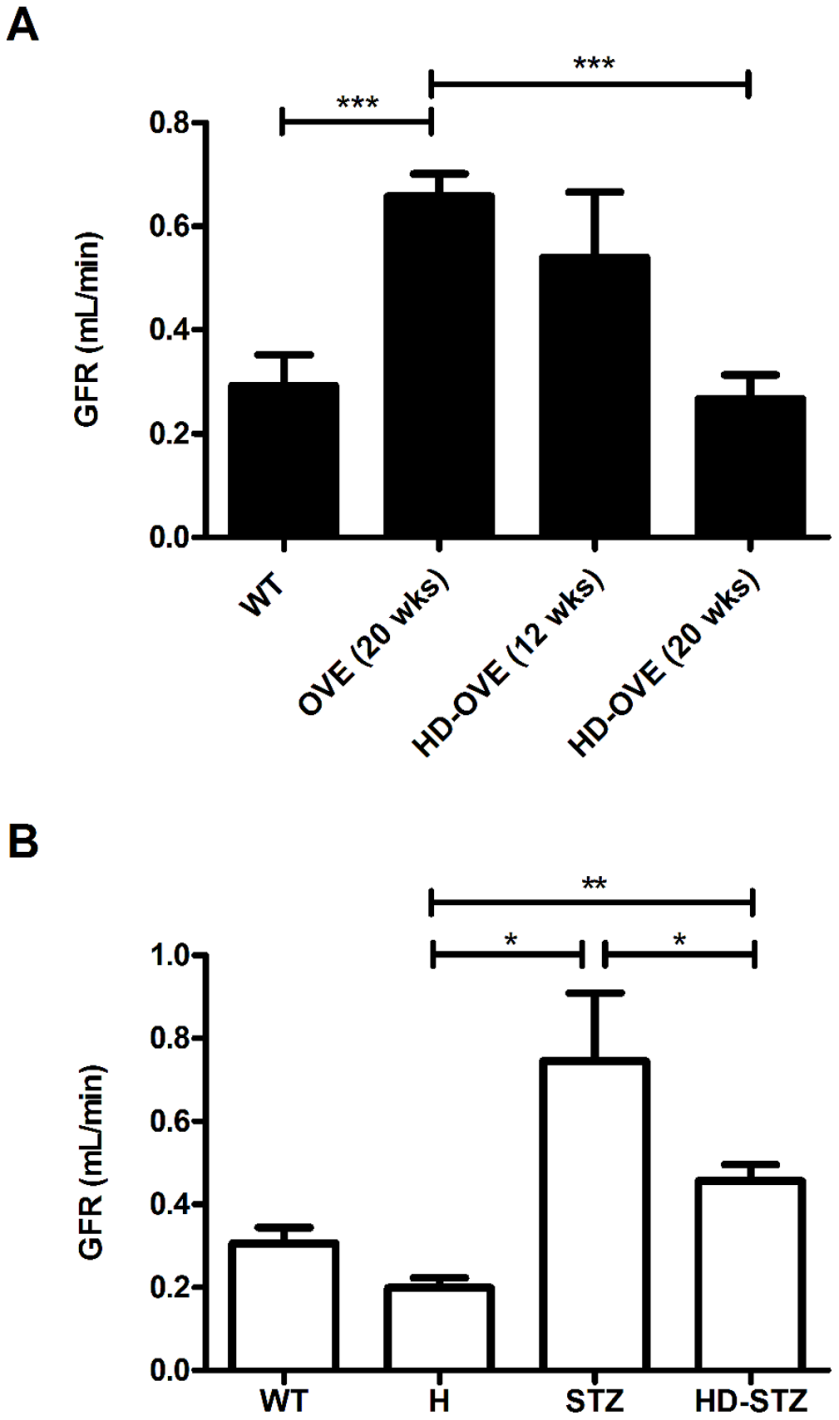
GFR estimation using FITC-inulin clearance. A) GFR was estimated in a subset of mice from the OVE26 study at early (12 weeks) and later (20 weeks) time points (2–6 mice/group) and B) in the STZ study at 18 weeks post-STZ (n = 5–9/group; * = P≤0.05; ** = P≤0.01; *** = P≤0.001).

As GFR decline is a key feature of late stage DN, we performed FITC-inulin GFR measurements in a subset of HD-OVE mice and at endpoint for the STZ study (Fig. 5A). Type 1 diabetic mouse models rarely show signs of renal function decline, and usually remain in the hyperfiltration stage [3]. HD-OVE mice exhibited hyperfiltration levels of GFR at 12 weeks of age, which were similar to levels seen in 20 week old OVE mice. By 20 weeks, HD-OVE mice showed significant GFR reductions compared to aged matched OVE mice, indicating a decline in renal function as disease progressed (20 weeks: OVE, 0.65±0.04; HD-OVE, 0.26±0.04, mL.min^−1^). Similarly, at 18 weeks post STZ, diabetes led to a 2-fold increase in GFR, while HD-STZ had significantly lower GFR values (WT, 0.31±0.04; H, 0.21±0.02; STZ, 0.75±0.15; HD-STZ, 0.45±0.04 mL.min^−1^).

## Discussion

Rodent models have provided important insights into the etiology of DN [10]. However, interpretations are tempered by the lack of an ideal model that reproduces not only early but also late characteristics of human DN [10], [11]. In the current report, we describe the generation of a novel DN model that addresses this concern by combining hypertension and diabetes (HD mice) resulting in an accelerated and robust nephropathy phenotype.

Provided they are bred onto so-called DN susceptible background strains (e.g., DBA/2, FVB/n, BLKS, etc.), the majority of currently available mouse models exhibit many of the characteristics of early DN [10], [11]. These include glomerular hyperfiltration, mesangial expansion, GBM thickening (>50% over baseline), glomerular and renal hypertrophy, arteriolar hyalinosis, and albuminuria. However, one or more key features of late DN are often absent – namely, GFR decline and/or tubulointerstitial fibrosis. Moreover, while hypertension often develops in humans as DN progresses [12], most rodent models exhibit limited increases in blood pressure (e.g., *Ins2^Akita/+^* mice, systolic blood pressure ∼130 mmHg [13]). A model that shows evidence of both early and late DN features is the OVE26 type 1 diabetic mouse. This line of transgenic mice was generated on the FVB/n background by Epstein *et al.* by overexpressing the calmodulin gene under the control of the rat insulin II promoter to allow for β-cell –specific expression [14]. Due to the destruction of the β-cells, OVE26 mice develop diabetes neonatally. FVB/n OVE26 mice exhibit many of the hallmarks observed in both early and late stage human DN [15]. These include an initial increase in GFR, accompanied by significant albuminuria. As the animals age, mesangial matrix expands, GBM thickens, tubulointerstitial fibrosis develops and kidney weight doubles. While GFR increases significantly early on in the OVE26 model, it declines between 5 and 9 months of age. Podocyte loss, a characteristic finding of human DN is evident after 16 months [16]. However, systolic BP changes minimally in OVE26 mice which may partly underlie the length of time needed for the DN phenotype to develop.

A model generated recently that features BP elevation is the *eNOS^−/−^* mouse [17], [18], [19]. Vascular endothelial nitric oxide synthase (eNOS) dimer formation and phosphorylation are reduced by high glucose in cultured endothelial cells suggesting impaired activity under diabetic conditions [20] - leading to attenuation of NO production and diminished vasodilatation. With increasing age, mice with targeted *eNOS* deletion subjected to low dose STZ-induced diabetes have normalized GFR, presumably due to a progressive decline in hyperfiltration, and exhibit tubulointerstitial fibrosis along with the onset of moderate hypertension [17], [18], [19]. *eNOS^−/−^* mice bred onto the type 2 diabetes *db/db* line which lack the leptin receptor exhibit even greater DN severity. Interestingly, recent studies by Harris’s group have underscored the importance of BP elevation for DN progression, in finding that glomerulosclerosis and albuminuria in *eNOS^−/−^ db/db* mice were decreased when BP was lowered independent of RAS inhibition [21].

However with many existing DN models, mice need to be of advanced age, some requiring 6–12 months for a consistent and full development of a DN phenotype [16]. Moreover, such models are limited by logistically challenging breeding strategies to arrive at(in some cases) triple homozygous compound gene-targeted animals. Together, these factors conspire to impede our ability to efficiently study the etiology of the disease. In light of these limitations, an accelerated and robust mouse model is needed for a more comprehensive understanding of diabetic nephropathy.

Our approach employs mice transgenic for the human renin cDNA under the control of the transthyretin promoter (TTRhRen) on an FVB/n background previously developed by Dr. Timothy Reudelhuber (U. of Montreal) [22]. Similar approaches have been realized by others using a variety of transgenes (i.e, RenTgARE, RenTgKC, and RenTgMK) on the 129S6/SvEvTac background [23], [24], [25]. A similar model was recently generated in rats, wherein the murine *renin-2* gene was driven by the cytochrome P450a1 promoter [26]. These rats become moderately hypertensive in response to indole-3-carbinol. Induction of hypertension along with STZ-induced diabetes produced a 500-fold increase in albuminuria, glomerulosclerosis and tubular interstitial fibrosis, while GFR tended to be lower in both diabetic and non-diabetic TTRhRen rats, but did not reach statistical significance. By translating a similar approach to mice using either STZ-induced or OVE26 type 1 diabetic mice, we have generated a model amenable to the current array of genetic strategies (i.e., gene-targeting/transgenics) that are used widely to explore the role of any number of putative players in the progression of DN. One caveat of the current approach is that unlike human diabetic nephropathy, where hypertension typically develops after indications of nephropathy have emerged, the HD model involves diabetes-induced renal injury with a concurrent elevation in blood pressure. Moreover, the HD mice do not represent non-proteinuric subsets of DN. Nevertheless, the HD mice developed in the present study fulfill much of the criteria set out by the Diabetes Complications Consortium [10], Specifically, both HD-STZ and HD-OVE mice have>10-fold increase in albuminuria, show evidence of widespread mesangial matrix expansion, and tubulointerstitial fibrosis. While tubular lesions appeared significantly more severe in HD-STZ vs. STZ mice, those which developed in HD-OVE mice represented even greater progression, perhaps due to the fact that the latter mice develop diabetes from a very early age. Following an initial period of hyperfiltration GFR declined progressively (by 50% of peak values) to levels within the ‘normal’ range for both HD-STZ and HD-OVE models. Given the extensive glomerular/tubular damage, it is likely that such a filtration rate represents hyperfiltration at the single nephron GFR level derived from residual glomerular function. Despite the presence of chronic hypertension, extensive glomerular and tubulointerstitial lesions in the HD models, we were unable to detect arteriolar hyalinosis. It remains possible that the relatively short duration of our models (<20 weeks) could account for the lack of this late human DN characteristic. We cannot therefore rule out whether arteriolar hyalinosis would have emerged if the mice were allowed to age beyond this time period. Additionally, while our model was successful on the FVB/n strain, whether it is amenable to more resistant strains (e.g., C57BL/6, which also become hypertensive with the TTRhRen transgene [27]) remains to be determined.

The accelerated phenotype of the HD model is likely due to superimposition of elevated blood pressure on a diabetic state. Both clinical and experimental data consistently show that interventions which reduce blood pressure are effective in mitigating renal disease progression in diabetes [28], [29], [30]. Indeed, blood pressure of HD-STZ mice was elevated in comparison to STZ mice alone, which did not differ from that of non-diabetic controls. In contrast, HD-OVE mice developed profound hypertension from 16–20 weeks of age (>180 mmHg) that dramatically exceeded that of non-diabetic renin-expressing mice. The underlying mechanism accounting for this difference is unclear. Despite these observations, one cannot discount blood pressure-independent effects of angiotensin II [31]. While we did not measure circulating or renal AngII in our HD models, previous studies showed plasma AngII in TTRhRen mice are 1–2 times normal [22] while renal levels are similarly elevated [32]. Such elevated AngII could exert damage-inducing effects directly upon the renal vasculature [21], glomerular filtration barrier [33], [34], [35] and tubular segments [13], [36]. Other transgenic models of hepatic renin overexpression, such as the RenTgMK mice (which show AngII levels 4-6-fold above wild type mice) exhibit glucose intolerance with normal fasting glucose levels and insulin sensitivity, suggesting that either circulating renin or AngII might impact glucose handling [37]. While we did not perform glucose tolerance tests on either TTRhRen or HD mice, blood glucose levels were invariably similar within non-diabetic or diabetic groups, suggesting that diabetes was induced equivalently irrespective of transgenic renin expression.

In summary, we have developed a mouse model of diabetic nephropathy with superimposed hypertension that recapitulates many key features of both early and late human disease over a relatively short timeframe. The HD model requires minimal breeding of readily available mouse lines and thus represents an attractive choice to study pathogenic mechanisms underlying diabetic nephropathy progression.

## Materials and Methods

### Physiological data

Blood samples were collected via cardiac puncture into heparinized syringes, kept on ice and centrifuged at 5000 g for 10 minutes at 4°C. Collected plasma was immediately frozen at −80°C until subsequent analysis. Plasma glucose levels were determined by glucometry (Bayer Contour). At sacrifice, tibias, kidneys and hearts were removed, individually weighed and organ weights were normalized to tibia length.

### Albuminuria

Albuminuria was measured using the Mouse Albumin Elisa Kit (Bethyl labs, Montgomery, TX.) following manufacturer’s protocol in spot urine samples. Albumin levels were determined by normalizing to creatinine concentration, determined by the Creatinine Companion kit (Exocell, Philadelphia, PA).

### Animals

Hypertensive TTRhRen mice have been previously described [32], [38]. Briefly, liver-specific expression of a modified human pro-renin cDNA transgene was achieved under control of a 3-kb region of the mouse transthyretin promoter. The synthesis of active human renin was optimized by introducing a furin cleavage site between the pro and active segments of the human renin transgene. Cleavage of the pro segment from the renin transgene occurs by the ubiquitously expressed furin enzyme in cells expressing this construct. Hyperreninemic TTRhRen mice on an FVB/N background display elevated systolic blood pressure (140–150 mmHg) and develop cardiac hypertrophy by 4 months of age [38] that may be attenuated by ACE inhibition or ARBs [27], [32], [38]. Hypertensive TTRhRen mice do not display a renal phenotype.

Hypertensive diabetic mice (HD) were generated using two type 1 diabetic mouse models including the streptozotocin (HD-STZ) and OVE26 (HD-OVE) models. The former was achieved using the low-dose STZ protocol [39]. Briefly, 8–10 week old wild-type (WT) or TTRhRen (H) male mice were subjected to 5-day intraperitoneal injections of STZ (50 mg kg^−1^ BW^−1^; Sigma-Aldrich, Oakville, ON.) or 0.1 M Na-Citrate buffer pH 4.5 as vehicle. The latter mouse model studied was the previously characterized transgenic OVE26 mice on the FVB/N background, which are insulinemic at birth due to pancreatic beta-cell specific overexpression of a calmodulin mini-gene [40]. HD-OVE mice were obtained by intercrossing OVE26 mice (Male, 2–3 months, Jackson Laboratory, Bar Harbor, ME) with TTRhRen mice (Female, 2–3 months). Experimental animals (male, 6–20 weeks) were housed and cared for in the Animal Care Facility at the University of Ottawa with free access to food and water. Protocols were approved by the University of Ottawa Animal Care Committee and conducted according to the guidelines of the Canadian Council on Animal Care.

### Blood pressure measurement

Throughout the study, systolic BP was measured via tail-cuff plethysmography (BP 2000, Visitech systems, Apex, NC) as described previously [32]. Daily systolic BP was calculated from measurements obtained at the same time period each day (5 preliminary, 10 actual BP readings/day) and, following a five-day training regimen (10 BP readings/day), weekly BP measurements were obtained.

### FITC-inulin clearance

Fluorescein isothiocyanate-labeled inulin (FITC-Inulin; Sigma-Aldrich, Oakville, ON.) clearance was used to estimate glomerular filtration rate (GFR). Briefly, 5% (w:v) FITC-inulin dissolved in 0.9% (w:v) saline was dialyzed (1000 MWCO) overnight and sterilized by filtration (0.2 µm). Anesthetized mice received a bolus (3.74 µL/g BW) of FITC-inulin via tail-vein injections. Blood samples (≈20 µL) were collected from the saphenous vein into heparinized capillary tubes, and centrifuged for 10 minutes at 10,000 RPM. Blood sampling was carried out at 3, 7, 10, 15, 35, 55 and 75 minutes post injection. Samples were buffered in 500 mM Hepes pH 7.4 and plasma fluorescence was measured (Excitation 488 nm/Emission 538 nm). A two-compartment clearance model was used to calculate GFR as previously described [41] using statistical analysis software (Graphpad Prism, San Diego, Ca.).

### Histology and α-SMA immunofluorescence

At sacrifice, mice were anesthetized (isoflurane), perfused with phosphate buffered saline (PBS) and kidneys were excised, dissected and immediately fixed in 4% paraformaldehyde (PFA). Paraffin-embedded kidney sections (3 µm) were obtained and stained with periodic-acid Schiff (PAS) or Masson’s Trichrome reagent. All sectioning, paraffin embedding and PAS-staining were performed by the University of Ottawa’s pathology department. Kidney sections were viewed under light-microscopy at either 200x or 400x magnification (Axioskop 2 Imager A1, Zeiss, Germany). Representative glomerular (20–25 glomeruli/mice) areas for each group were analyzed in a blinded manner. Imaging software (Axiovision v4.8, Carl Zeiss, Germany) was used to calculate relative mesangial matrix/glomerular area, whereby the area of the mesangial scar as a percentage of total glomerular area was determined.

Kidney α-smooth muscle actin (α-SMA; Santa Cruz Biotechnology, Dallas, TX.) immunofluorescence was performed on paraffin-embedded sections mounted on glass slides. Sections were deparaffinized in mixed xylenes (Fisher), and rehydrated through a gradient of ethanol and distilled water. Sections were washed 3x in PBS, boiled for 20 minutes in 0.1 M Na-citrate buffer (pH 6.0) for antigen unmasking. Sections were blocked in PBS containing 10% donkey serum/1% BSA for 1 hour and incubated with mouse anti-α-smooth muscle actin (1∶200) overnight at 4°C. Slides were washed and treated with a FITC-labelled donkey anti-mouse secondary antibody (1∶1000; Molecular Probes, Burlington, ON.) for 1 hour, followed by 4,6-diamidino-2-phenylindole (DAPI; Sigma-Aldrich, Oakville, ON.) for nuclear localization. Sections were covered with fluorescent mounting medium (Vector laboratories, Burlington, ON.) and coverslips. Slides were visualized under fluorescence microscopy whereby representative cortical profiles from each group were obtained in a blinded manner.

### Western immunoblotting and quantitative PCR

Cortical kidney tissue was homogenized with a COE Capmixer and suspended in RIPA lysis buffer (150 mM NaCl, 1% Triton X-100, 0.5% sodium deoxycholate, 0.1% SDS and 50 mM Tris pH 8.0), supplemented with protease inhibitor cocktail 1∶100 (Sigma-Aldrich, Oakville, ON). Protein lysates were processed by SDS-PAGE, transferred to nitrocellulose membranes, incubated with appropriate antibodies and processed for chemiluminescence. Primary antibodies, including rabbit anti-fibronectin 1∶1000 (Sigma-Aldrich, Oakville, ON) and mouse anti-α-tubulin 1∶2000 (Sigma-Aldrich, Oakville, ON) were incubated o/n at 4°C. Secondary antibodies, including HRP-goat anti-rabbit 1∶10000 (Jackson ImmunoResearch Laboratories, West Grove, PA) and HRP-goat anti-mouse 1∶10000 (Jackson ImmunoResearch Laboratories, West Grove, PA), were incubated for 1 hour at room temperature. For quantitative PCR (qPCR), kidney tissue was homogenized using QIAshredder columns (Qiagen). RNA was extracted using the Qiagen RNEasy minikit as per manufacturer’s instructions. Extracted RNA was converted to cDNA using the High-Capacity cDNA Reverse Transcription kit (Applied Biosystems) with 500 ng starting material per reaction. Assay was performed using an ABI Prism 7000 Sequence Detection System with SYBR Advantage qPCR Premix (Clontech) according to manufacturer’s instructions. Primers used: Collagen-IV sense (5′- ATGGGGCCCCGGCTCAGC −3′), Collagen-IV antisense (5′- ATCCTCTTTCACCTTTCAATAGC −3′); GAPDH sense and antisense were purchased from Invitrogen.

### Statistical analysis

The values are presented as means ± SE. Statistical comparisons between two-groups was performed using the unpaired Student’s *t*-test, while analysis of variance (ANOVA) was used for three or more groups, followed by a Newman-Keuls post-test. Statistical significance was achieved when P≤0.05.
